# Allosteric Modulators of Class B G-Protein-Coupled Receptors

**DOI:** 10.2174/157015907781695928

**Published:** 2007-09

**Authors:** Sam R.J Hoare

**Affiliations:** Department of Discovery Biology, Neurocrine Biosciences Inc., San Diego, California, USA

**Keywords:** Allosteric, class B, G-protein-coupled receptor, secretin, corticotropin-releasing factor, nonpeptide, glucagon-like peptide, parathyroid hormone.

## Abstract

Class B GPCR’s are activated by peptide ligands, typically 30-40 amino acid residues, that are involved in major physiological functions such as glucose homeostasis (glucagon and glucagon-like peptide 1), calcium homeostasis and bone turnover (parathyroid hormone and calcitonin), and control of the stress axis (corticotropin-releasing factor). Peptide therapeutics have been developed targeting these receptors but development of nonpeptide ligands, enabling oral administration, has proved challenging. Allosteric modulation of these receptors provides a potential route to developing nonpeptide ligands that inhibit, activate, or potentiate activation of these receptors. Here the known mechanisms of allosteric modulators targeting Class B GPCR’s are reviewed, particularly nonpeptide antagonists of the corticotropin-releasing factor 1 receptor and allosteric enhancers of the glucagon-like peptide-1 receptor. Also discussed is the potential for antagonist ligands to operate by competitive inhibition of one of the peptide binding sites, analogous to the Charniere mechanism. These mechanisms are then used to discuss potential strategies and management of pharmacological complexity in the future development of allosteric modulators for Class B GPCR’s.

## INTRODUCTION

The class B G-protein-coupled receptor (GPCR) family is a small group of receptors, 15 in the human genome, that are activated by intermediate sized peptides of typically 30-40 amino acid residues (Table **1**) [17, 21]. These peptides mediate a diverse array of important homeostatic processes and other physiological functions, acting as hormones, autocrine factors and neuromodulators (Table **1**). For example, parathyroid hormone and calcitonin reciprocally regulate calcium homeostasis and bone turnover, activating PTH1 and calcitonin receptors, respectively [54, 66]. Glucagon regulates hepatic glucose output [45, 52, 77], and post-prandial glucose homeostasis is modulated by the incretin peptides glucagon-like peptide-1 (GLP-1) and glucose-dependent insulinotropic peptide (GIP) [18, 52, 77]. Corticotropin-releasing factor is the principal regulator of the stress axis, acting peripherally *via* activation of the hypothalamic-pituitary-adrenal (HPA) axis and centrally by means of modulating behavioral responses to stress [2, 20, 32, 81]. 

At least eight of the fifteen human Class B GPCR’s have received attention as potential targets for the treatment of disease (Table **1**). In some instances therapeutic agents have been developed from the peptides themselves. Calcitonin and parathyroid hormone are in clinical use for treatment of osteoporosis [54, 58]. A reptilian analogue of GLP-1, Exanatide (Byetta), has recently been developed as a mechanistically novel therapeutic for the management of Type 2 diabetes [15, 19]. The major physiological and therapeutic function of these peptides has stimulated considerable understanding of their receptor binding and receptor activation mechanisms. Intrinsic to these mechanisms for Class B GPCR’s is the potential for allosteric regulation by nonpeptide ligands, which could aid the development of orally bioavailable modulators of Class B GPCR’s to circumvent practical and potential compliance issues with injections of the peptide therepeutics. Also intrinsic to the peptide binding mechanism is the potential for a modulator to act by competitive blockade of one of the peptide binding sites, a mechanism analogous to the Charniere model [69]. This mechanism challenges the utility of commonly-used analytical pharmacology methods to precisely define the mechanism of action of the modulator. Here the peptide binding mechanisms are briefly reviewed, the modulator mechanisms evaluated with reference to specific examples, and then this information is applied to discuss methods and analytical issues in the future development of nonpeptide allosteric ligands targeting Class B GPCR’s.

## TWO-DOMAIN MODEL OF PEPTIDE LIGAND INTERACTION WITH CLASS B GPCR’S

Class B GPCR’s likely share a similar secondary and tertiary structure [17]. The extracellular N-terminal region, herein termed the N-domain, comprises approximately 100-160 amino acid residues. The NMR structure of the N-domain of the CRF_2(b)_ receptor indicates a folded structure stabilized by three disulfide bonds, a hydrophobic core and an internal salt bridge [25]. The folded structure is comprised of β-sheets in an orientation that is described as a short consensus repeat, or Sushi domain [25, 62]. The key residues involved in maintaining this structure are highly conserved throughout the Class B GPCR family, suggesting this structure is common to all Class B GPCR’s [25]. The remaining juxtamembrane domain of the receptor (J-domain) comprises seven predicted membrane-spanning α-helices with intervening intracellular and extracellular loops [17]. The structure of the J-domain has not been determined directly. It shares little primary structural homology with rhodopsin (for which the X-ray structure has been determined [60]) but mutagenesis and zinc-bridging studies suggest certain tertiary structure-stabilizing elements might be similar between Class A and Class B GPCR’s [22, 76].

**Fig. (1):**
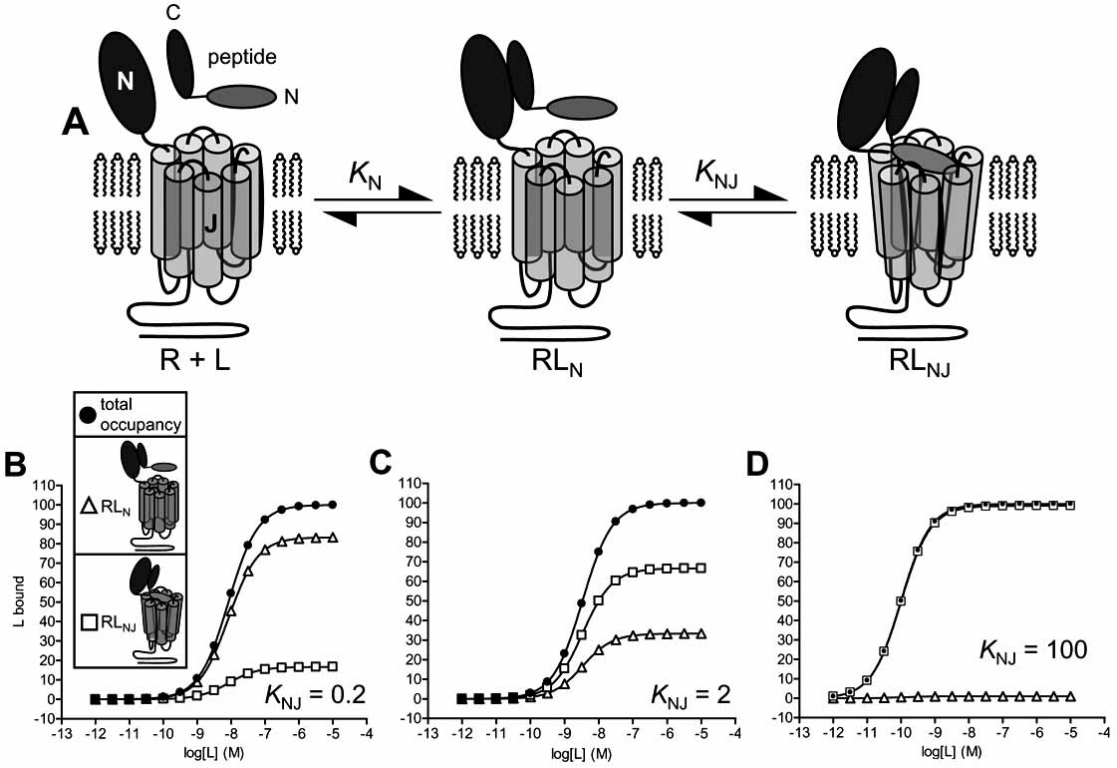
**Two domain model of peptide interaction with Class B GPCR’s.** A. Schematic representation of the two-domain model. The carboxyl-terminal portion of the peptide binds the extracellular N-terminal domain (N-domain) of the receptor, forming RL_N_, defined by the equilibrium constant *K*_N_. This interaction acts as an affinity trap, promoting interaction of the amino-terminal portion of the ligand with the juxtamembrane domain (J-domain) of the receptor, forming RL_NJ_. The J-domain interaction is defined by the isomerization constant *K*_NJ_, defining the RL_NJ_ : RL_N_ concentration ratio. B-D: Simulation of receptor occupancy, N-domain occupancy and J-domain occupancy by peptide ligand with varying strength of J-domain interaction. Occupancy of the receptor was defined by eq. 1 (Appendix), occupancy of the N-domain alone by eq. 2, and occupancy of the J-domain (with concomitant occupancy by the N-domain) by eq. 3. The parameter values used were: *K*_N_ = 1×10^8^ M^-1^, [R]_TOTAL_ = 100, and values of *K*_NJ_ of 0.2 (B, weak J-domain interaction), 2 (C, moderate interaction)and 100 (D, strong interaction).

The orientation and mechanism of peptide interaction with Class B GPCR’s has been studied extensively using peptide structure-activity relationships (SAR) [7, 46, 79], receptor and ligand fragments [1, 38, 49, 61, 63, 79], chimeric receptors [3, 41, 74], site-directed mutagensis [33, 46], photochemical cross-linking [11, 16, 51, 65], NMR structure determinations [4, 25, 55, 62] and molecular modeling [4, 11, 25, 65, 72]. Almost all of the data are consistent with a low-resolution molecular orientation of binding in which the carboxyl-terminal portion of the peptide binds to the N-domain of the receptor and the amino-terminal portion of the peptide binds and activates the J-domain (Fig. **1A**). For example, a chimeric peptide formed of the carboxyl-terminal portion of calcitonin and the amino-terminal portion of parathyroid hormone activates a chimeric receptor comprising the N-domain of the calcitonin receptor and J-domain of the PTH1 receptor [3]. The same orientation was inferred using the reciprocal chimeras [3], and with glucagon/GLP-1 ligand and receptor chimeras [74]. The carboxyl-terminal portion of the peptides typically form an α-helix in binding to the N-domain of the receptor [55]. This interaction is of moderate-to-high affinity (1-100nM) [1, 36, 38, 39, 61] and does not appear to be directly involved in receptor activation [14]. As a result, carboxyl-terminal fragments act as high-affinity antagonists [49, 68, 87]. Interaction between the amino-terminal region of the peptide with the J-domain is of much lower affinity (in the high μM range). This interaction activates the receptor, stimulating intracellular signaling [50, 59, 78, 79]. Class B GPCR’s signal predominantly through G_S_-coupled pathways and, to a more limited extent, through G_q/11_ and G_i_ family G-proteins.

The two-domain model provides a simple and tractable mechanistic framework for interpreting peptide and allosteric modulator binding mechanisms [1, 33, 79]. One formulation of the model is presented in Fig. (**1A**). Peptide ligand binds the receptor N-domain, defined by the equilibrium association constant *K*_N_. This interaction provides an affinity trap, increasing the local concentration of the amino-terminal portion of the peptide in the vicinity of the J-domain, overcoming the low-affinity of this interaction to enable significant binding to occur. This binding event is represented here as an isomerization between RL_N_, ligand bound to the N-domain alone, and RL_NJ_, ligand bound to both N- and J-domains (Fig. **1A**). The strength of this interaction is described here by an isomerization constant, *K*_NJ_, representing the RL_NJ_ : RL_N_ concentration ratio at equilibrium.

In order to understand mechanisms of allosteric modulation, it is instructive to consider the extent of peptide occupancy of N- and J-domains, under different conditions, for different receptors, and for different peptides binding the same receptor. In Fig. (**1A**), ligand occupancy of the N-domain alone is represented by RL_N_, and ligand occupancy of the J-domain is represented by RL_NJ_. The extent of receptor occupancy by the N-domain is largely dependent on the peptide concentration and *K*_N_. The fraction of peptide-bound receptors in which peptide is bound to the J-domain is dependent on *K*_NJ_. This binding energy varies between Class B GPCR’s and is dependent on the conformational state of the receptor. (Similar to most Class A GPCR’s the conformational state of Class B GPCR’s regulated by receptor-G-protein interaction [71], reflected in the higher agonist affinity at the G-protein coupled state (RG) compared with the uncoupled state (R).) At the R state, the inferred value of *K*_NJ_ is quite low for some Class B GPCR’s (e.g. <1 at PTH1 and CRF_1_ receptors [35, 38]). Under these conditions, the majority of peptide-occupied receptors have ligand bound only to the N-domain (Fig. **1B**), with only a small fraction having ligand bound to J-domain (Fig. **1B**). Importantly, under these conditions peptide ligand cannot saturate the J-domain of the receptor, even at ligand concentrations that saturate the receptor through occupancy the N-domain (Fig. **1B**). Consequently, even at saturating peptide concentrations unoccupied J-domain will be available for other ligands to bind to, a significant property in understanding modulator mechanisms (see below). Slightly stronger interaction with the J-domain (*K*_NJ_ = 2) increases the fraction of occupied receptors in which peptide is bound the J-domain (Fig. **1C**). Finally for some receptors a high value of *K*_NJ_ has been inferred at the R state (e.g. 30 for urocortin 2 interaction with the CRF_2_ receptor [36]). Under these conditions peptide is bound to the J-domain in almost all ligand-occupied receptors (Fig. **1D**). G-protein interaction with the receptor likely increases the strength of ligand binding to the J-domain, which can be represented by an increase of *K*_NJ_ [38, 39]. As evident from comparing Fig. (**1B-D**), this effect of G-protein increases the fraction of occupied receptors in which ligand is bound to the J-domain. This inference is important in comparing modulator actions at R and RG states (see below).

## ALLOSTERIC MODULATORS OF THE CRF_1_ RECEPTOR

**Fig. (2):**
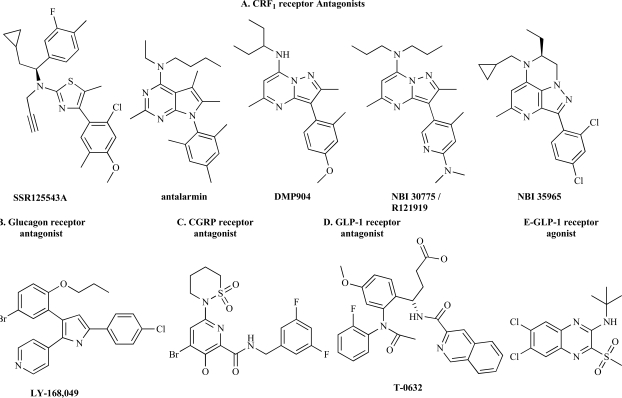
**Chemical structure of allosteric modulators of Class B GPCR’s.** A. CRF_1_ receptor antagonists (SSR125543A [27]; antalarmin [89]; DMP904 [23]; NBI 30775 [8]; NBI 35965 [29]). For a review of CRF_1_ receptor antagonist chemical structure see ref [43]. B. Glucagon receptor antagonist [6, 13]. C. CGRP antagonist [75]. D. GLP-1 receptor antagonist [86]. E. GLP-1 receptor agonist [44].

Allosteric modulators have been identified and developed for numerous Class A and Class C GPCR’s (see reviews in this issue). Mechanistic studies including analytical pharmacology, ligand SAR and receptor modification have elaborated general concepts and specific mechanisms of how allosteric ligands produce their modulatory effects. These methods have been applied to identify allosteric modulators of Class B GPCR’s. Low-molecular weight, nonpeptide allosteric modulators have been identified for CRF_1_, glucagon and GLP-1 receptors (Fig. **2**) [6, 24, 43-45, 75, 80, 86]. Mechanistic studies, particularly with the CRF_1_ receptor, have demonstrated that knowledge of the mechanism of peptide binding is highly useful in elaborating the mechanisms of allosteric modulation of Class B GPCR’s.

Allosteric modulators of the CRF_1_receptor have been studied in the most detail, in terms of ligand SAR [24, 43, 53], *in vivo* efficacy [28] and allosteric mechanism of action. Antagonism of central CRF_1_ receptors has been proposed as a potential novel mechanism for the treatment of anxiety, depression and other stress-related disorders, such as irritable bowel syndrome [28, 40, 56, 85]. This proposal has stimulated the discovery and development of a broad array of orally-available, CNS-penetrating nonpeptide antagonists that bind with high affinity (low nonomolar) to the CRF_1_receptor. Prototypical examples include CP-154,526 [9], antalarmin [89], DMP696 [31], DMP904 [23], SR125543A [27] and NBI 30775 [8] (also known as R121919) (Fig. **2**). Nonpeptide antagonists are active in animal models of CRF- and environmentally-induced responses to stress [24, 28, 43, 53]. NBI 30775 has been tested in human subjects. This compound significantly reduced Hamilton depression and anxiety scores in severely depressed individuals in a small open-label Phase IIa clinical trial [92].

The first evidence that nonpeptide antagonists of the CRF_1_ receptor act allosterically was provided by receptor mutation studies to identify the ligand binding site [48]. Mutation of two residues within the predicted membrane-spanning region of the receptor (H199V and M276I) reduced binding of the nonpeptide antagonist NBI 27914 without affecting binding of peptide agonists (e.g. CRF). This finding suggests the binding sites for nonpeptide antagonist and peptide ligand are at least partially distinct. This hypothesis is supported by subsequent findings that strongly imply M276 is proximal to the bound nonpeptide ligand [34]. In addition, the peptide binding determinants that have been identified to date are located within extracellular regions of the receptor – the N-domain and the extracellular loops of the J-domain (reviewed in refs [12, 25, 34, 62]. Taken together these findings suggest CRF_1_ receptor nonpeptide antagonists bind within the membrane-spanning region of the J-domain and peptide ligands bind to sites further towards the extracellular face of the receptor, implying allostetric interaction between peptide and nonpeptide ligand.

Radioligand binding studies are consistent with an allosteric interaction between nonpeptide antagonist and peptide ligands at the CRF_1_ receptor [37, 91]. In radioligand dissociation assays, nonpeptide ligands modulate the dissociation of radiolabeled peptides from the receptor and, reciprocally, peptide ligands modulate dissociation of radiolabeled nonpeptides [37]. In equilibrium binding assays, peptide ligands do not fully inhibit specific binding of radiolabeled nonpeptides [37, 91]. Nonpeptide ligands decrease the apparent affinity of peptide ligands but this decrease of affinity approaches a limit as the concentration of nonpeptide ligand increases [37]. All of these features are consistent with the allosteric ternary model described for Class A GPCR’s such as muscarinic acetylcholine receptors [47, 84]. In this model, modulator can bind to the receptor occupied by endogenous ligand, and vice versa, forming a ternary complex between receptor, modulator and endogenous ligand.

**Fig. (3):**
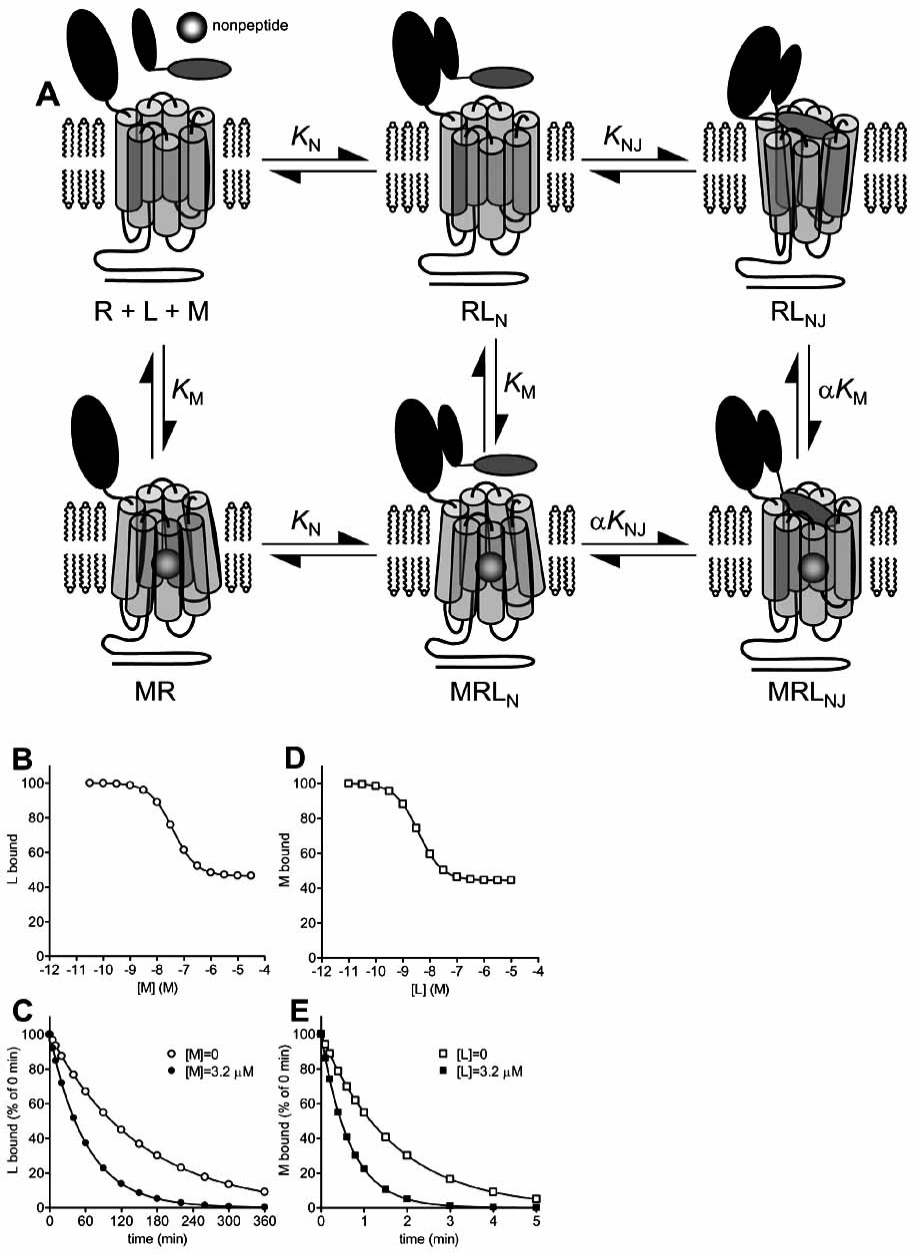
**Allosteric modulation of peptide binding to Class B GPCR’s *via* the J-domain.** A. Schematic representation of the model. Peptide binding is described by the two domain model (Fig. **1A**). Allosteric modulator binds the J-domain of the receptor at a site spatially distinct from the peptide binding regions, defined by the equilibrium constant *K*_M_. Binding of modulator allosterically regulates binding of peptide to the J-domain, defined by the cooperativity factor α, without affecting peptide binding to the N-domain. B-E. Manifestation of the model in binding assays for an allosteric inhibitor, simulated using equations in the Appendix. Binding parameters were: *K*_N_ = 1×10^8^ M^-1^, [R]_TOTAL_ = 100, *K*_NJ_ = 2, *K*_M_ = 1×10^8^ M^-1^ and α = 0.1 (allosteric inhibition, negative cooperativity). B. Modulation of equilibrium binding of peptide (L) by modulator (M), simulated using eq. 5, [L] = 1×10^-9^ M. C. Modulation of peptide dissociation by modulator, simulated using eq. 7. The dissociation rate of peptide from the N-domain (*k*_-N_) was set at 0.02 min^-1^. D. Modulation of equilibrium binding of modulator by peptide ligand, simulated using eq. 8, [M] = 2×10^-9^ M. E. Modulation of modulator dissociation by peptide, simulated using eq. 12. The dissociation rate of modulator (*k*_-M_) was set at 0.6 min^-1^ and the dissociation rate of modulator from MRL_NJ_ (*k*_-M(L)_) was set at 6.0 min^-1^.

The peptide-receptor interactions that are modulated by nonpeptide antagonists have been studied using receptor and peptide fragments [37, 38, 59, 64]. Binding of peptide agonists to the CRF_1_ receptor is well-described by the two domain model described above and illustrated schematically in Fig. (**1A**) [25, 38, 64]. Nonpeptide binding determinants are borne largely if not exclusively by the J-domain;. nonpeptide antagonist affinity for a J-domain fragment is not significantly different from that for the full-length receptor and the ligands do not displace radiolabed peptide binding to a N-domain fragment [38]. The peptide interaction that is blocked by nonpeptide antagonists appears to be that between the amino-terminal region of the peptide and the J-domain of the receptor. Binding of a radiolabeled amino-terminal-truncated peptide ([^125^I] astressin, a CRF(12-41) analogue) to the wild-type CRF_1_ receptor is not appreciably inhibited by nonpeptide ligands [37, 38, 64], and nonpeptide antagonists completely block CRF-stimulated activation of a J-domain fragment [38, 59]. Taken together these findings are consistent with the model in Fig (**3A**). In this model, CRF binding is described by the two-domain mechanism. Nonpeptide ligand binds within the membrane-spanning region of the J-domain, a site distinct from the CRF binding regions located further towards the extracellular face of the J-domain (Fig. **3A**). Nonpeptide binding does not affect CRF binding to the N-domain, but allosterically inhibits CRF binding to the J-domain. Since CRF interaction with the J-domain is required for receptor activation, blocking this interaction with nonpeptide ligand effectively antagonizes CRF-stimulated signaling. The behavior of this model, an extended variant of the allosteric ternary model, was simulated and rationalized as described in the Appendix and presented in Fig. (**3B-E**). In these simulations, nonpeptide ligand does not fully inhibit equilibrium binding of peptide ligand (Fig. **3B**) and reciprocally peptide does not fully inhibit binding of nonpeptide (Fig. **3C**). In radioligand dissociation experiments nonpeptide modulates dissociation of peptide ligand (Fig. **3D**), and vice versa (Fig. **3E**).

This model can explain an interesting feature of nonpeptide modulator action at the CRF_1_ receptor. In inhibiting peptide binding, nonpeptide ligands display a much greater allosteric effect (negative cooperativity) at the G-protein-coupled state of the receptor (RG) compared with the uncoupled state (R) [37]. Nonpeptides near-fully inhibit peptide binding to the RG state but only partially inhibit peptide binding to R. This effect can be explained by a stronger peptide-J-domain interaction at the RG state compared with the R state. CRF binds with moderate affinity to the N-domain (approximately 50nM [38, 61]). At the R state, interaction with the J-domain is weak (*K*_NJ_ <1), whereas at the RG state the J-domain interaction is much stronger (*K*_NJ_ >100) [38]. Consequently the extent of peptide occupancy of the J-domain is predicted to differ dramatically between R and RG states, with low J-domain occupancy at the R state (e.g. Fig. **1B**) and high occupancy at the RG state (e.g. Fig. **1D**). Consequently, inhibition of J-domain occupancy by nonpeptide ligand does not dramatically affect total receptor occupancy by peptide at the R state, because only a minor fraction the occupied receptors has peptide bound to the J-domain (Fig. **1B**). In contrast, at the RG state, inhibition of J-domain occupancy by nonpeptide ligand strongly reduces total receptor occupancy by peptide because almost all of the occupied receptors have peptide bound to the J-domain (Fig **1D**).

## NONPEPTIDE ALLOSTERIC MODULATORS OF OTHER CLASS B GPCR’S

Nonpeptide antagonists have been identified for glucagon [6, 45], CGRP [75] and GLP-1 receptors [44, 86] that have been shown or inferred to act allosterically (Fig. **2B-E**). The mechanisms by which these ligands modulate their receptors have not been established in detail. Antagonism of the glucagon receptor has received interest as a potential mechanism for managing hyperglycemia in the treatment of Type 2 diabetes [45]. Numerous structural classes of high-affinity nonpeptide antagonists have been identified, with examples including L-168,049 (Fig. **2B**), Bay 27-9955 and NNC 25-2504 [45]. L-168,049 has been shown to allosterically modulate glucagon interaction with the glucagon receptor [6]. The ligand slows dissociation of [^125^I]glucagon, and two mutations within the membrane-spanning region of the receptor reduce affinity of the antagonist without affecting binding of glucagon. These findings are potentially consistent with the ligand acting by a similar allosteric mechanism as that identified for nonpeptide antagonists of the CRF_1_receptor (Fig. **3A**). By contrast, a nonpeptide antagonist of the GLP-1 receptor, T-0632 (Fig. **2D**), modulates the receptor by a different mechanism [86]. This low-affinity (1μM) ligand binds the N-domain of the GLP-1 receptor, at a site that appears at least partially distinct from the peptide binding sites; W33S mutation in the N-domain decreases T-0632 affinity 120-fold without appreciably affecting binding of peptide ligand. Finally, a low-affinity (3μM) nonpeptide antagonist of the CGRP receptor (Fig. **2C**) has been inferred to act allosterically from studies of chimeric receptors [75].

Recently, the first allosteric agonist of a Class B GPCR was reported [44]. A representative of the compound class is shown in Fig. (**2E**). This nonpeptide ligand enhances the binding of [^125^I]GLP-1 to the GLP-1 receptor and directly activates the receptor, with potency of approximately 100nM. This direct agonist activity is apparent for the receptor expressed endogenously and is GLP-1 receptor mediated: The modulator stimulates insulin release from pancreatic islets and perfused pancreas, and does not affect insulin release from islets isolated from GLP-1 receptor knockout mice [44]. Although the modulator enhances the binding of GLP-1 it does not detectably enhance the signaling activity of the peptide. 

## POTENTIAL FOR ‘CHARNIERE’ TYPE MODULATION OF CLASS B GPCR’S

Inherent to the two-domain mechanism of peptide binding to Class B GPCR’s is a modulatory mechanism that can appear allosteric but which actually arises from competitive inhibition at one of the peptide binding sites. An explicit description of this type of phenomenon is the ‘Charniere’ effect [69]. In this mechanism an antagonist bears two functional groups, connected by a hinge-region, one that binds the agonist binding site and the other that binds a distinct site not bound by the agonist. This model was developed to explain two unusual actions of some antagonists – persistent blockade after washout and subsequent treatment with agonist, and a time-course of the subsequent agonist response that is independent of the concentration of agonist [69, 70]. This model was applied to explain blockade of histamine responses [70], and blockade of acetylcholine by lachesine at muscarinc receptors of guinea pig ileum [69], and has been subsequently applied to other receptor systems, such as blockade of angiotensin-stimulated tachyphylaxis of rat uterine smooth muscle by a chlorambucil-substituted peptide antagonist [83]. The Charniere concept can be expanded to include persistently-binding large agonists, which are blocked at their readily-reversible site of interaction by smaller antagonists. For example, salmeterol is a β_2_ adrenergic agonist that binds the endogenous-ligand binding site, blocked by classical antagonists, and a second ‘exosite’ to which it persistently binds and which is not blocked by antagonists [26, 42]. A similar mechanism has also been used to explain the characteristics of xanomeline receptor interaction with the M_1_ muscarinc acetylcholine receptor [10]

**Fig. (4):**
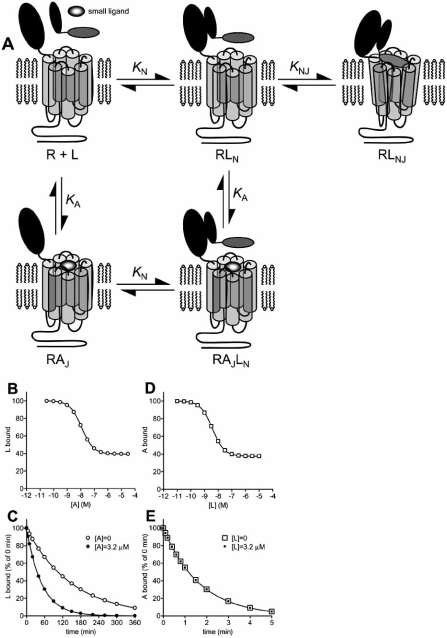
**Charniere-type model of modulator interaction with Class B GPCR’s.** A. Schematic representation of the model. In this model, a small ligand binds to one of the two sites of peptide interaction, in this specific example the J-domain. Binding of small ligand to this site, defined by the equilibrium constant *K*_A_, competitively inhibits peptide interaction with the J-domain but does not affect peptide binding to the N-domain. Note that in this model there is no allosteric interaction between the small ligand and peptide. B-E. Manifestation of the model in binding assays, simulated using equations in the Appendix. Binding parameters were: *K*_N_ = 1×10^8^ M^-1^, [R]_TOTAL_ = 100, *K*_NJ_= 2, and *K*_A_ =1×10^8^. B. Modulation of equilibrium binding of peptide (L) by small ligand (A), simulated using eq. 13, [L] = 1×10^-9^ M. C. Modulation of peptide dissociation by small ligand, simulated using eq. 15. The dissociation rate of peptide from the N-domain (*k*-_N_) was set at 0.02 min^-1^. D. Modulation of equilibrium binding of small ligand by peptide ligand, simulated using eq. 17, [A] = 2×10^-9^ M. E. Modulation of small ligand dissociation by peptide, simulated using eq. 19. The dissociation rate of small ligand (*k*_-A_) was set at 0.6 min^-1^. Note that this model does not allow peptide to modulate dissociation of small ligand, in contrast to the allosteric model (Fig. **3E**).

Intrinsic to the two domain model is the potential for a Charniere-type modulation because a small ligand could competitively inhibit peptide binding to one of the two domains without affecting peptide interaction with the other. The behavior of one potential mechanism within this general model was evaluated. In Fig. (**4A**), a small ligand binds the J-domain at the same site that binds the amino-terminal region of peptide ligand, competitively inhibiting peptide-J-domain interaction. The small ligand does not affect binding of the carboxyl-terminal portion of the peptide to the N-domain of the receptor. Importantly, in this model there are no allosteric interactions between the small ligand and the peptide. Fig. (**4B-E**) and the Appendix describe the manifestation of this model in ligand binding experiments typically used to identify allosteric modulators. In this simulation it was assumed the strength of peptide binding to the J-domain was moderate (*K*_NJ_= 2), such that occupancy of the J-domain represented a significant fraction of total receptor occupancy by peptide ligand (Fig. **1C**). Under these conditions, the small ligand inhibits binding of the peptide but saturating concentrations of the small ligand only partially inhibit peptide binding (Fig. **4B**). The residual receptor occupancy by peptide is comprised of peptide bound only to the N-domain of the receptor. In peptide ligand dissociation experiments, the small ligand can accelerate dissociation of the peptide ligand (Fig. **4C**). This effect is a result of peptide only dissociating from the RL_N_ state, rather than the RL_NJ_ state, in the model. Dissociation of peptide from RL_N_ is slowed by formation of RL_NJ_. The small ligand inhibits peptide binding to the J-domain of the receptor, preventing the formation of RL_NJ_ that slows peptide dissociation, and consequently accelerating dissociation of peptide. In Fig. (**4D**), peptide inhibits small ligand binding but saturating concentrations of peptide do not fully inhibit small ligand binding. This effect is a result of the only partial occupancy of the J-domain by peptide ligand at saturating concentrations of peptide ligand (Fig. **1C**); the remaining receptors are available to be bound by the small ligand. Finally, in dissociation experiments peptide ligand cannot affect the observed dissociation rate of the small ligand (Fig. **4E**). The presence of pre-bound small ligand prevents binding of the peptide ligand to the J-domain, and peptide binding to the N-domain does not affect dissociation of the small ligand.

It is instructive to compare the potential consequences of this model, in which no allosteric interaction is involved, with the allosteric model presented in Fig. **3**. The models are similar in that the modulating ligand binds the J-domain of the receptor but they differ in the mechanism by which the modulating ligand inhibits J-domain binding of the peptide (competitive in the direct interaction model, Fig. **4A**, allosteric in the allosteric model, Fig. **3A**). Both mechanisms can result in partial inhibition of equilibrium binding of peptide (Figs. **3B** and **4B**), acceleration of peptide dissociation from the receptor (Figs. **3C** and **4C**) and partial inhibition of modulator binding by the peptide (Figs. **3D** and **4D**). Consequently, identifying these patterns of behavior in binding assays is potentially insufficient, in the absence of other data, to define a ligand as an allosteric modulator. The principle difference between the models is that peptide ligand can modulate dissociation of an allosteric modulator, but not a competitive inhibitor of a peptide binding site. (Fig. **3E** and **4E**). In addition, two other results are only possible with the allosteric mechanism – enhanced binding of peptide by modulator (and vice versa), resulting from positive cooperativity, and slowing of peptide dissociation by modulator (see Appendix). These considerations suggest that, for Class B GPCR’s, care should be employed in the interpretation of results from binding experiments typically used to define a compound as an allosteric modulator. As described below, mischaracterizing the mechanism of action of a ligand could impact further ligand optimization.

The potential ambiguity between these mechanisms has arisen at least twice in the literature. In the first case, certain characteristics of nonpeptide antagonists of the CRF_1_ receptor could potentially be explained by both mechanisms; nonpeptide antagonists only partially inhibit peptide ligand binding, and peptides only partially inhibit radiolabeled nonpeptide ligand binding [37]. Defining these compounds as acting allosterically was possible because the peptide ligands modulate radiolabeled nonpeptide dissociation from the receptor, nonpeptides slow dissociation of radiolabeled peptides [37], and perhaps most importantly mapping the binding sites by site-directed mutagenesis identified them as being spatially distinct on the receptor [34, 48]. In the second case, an amino-terminal analogue of parathyroid hormone (a modified PTH(1-14) fragment) only partially displaced binding of [^125^I]PTH(3-34) [35]. Reciprocally, PTH(1-34) only partially inhibited interaction of the PTH(1-14) analogue. This interaction between the two ligands was formerly described as allosteric [35] but in the absence of ligand dissociation data this conclusion cannot be reliably drawn. The model described in Fig. (**4A**) is a more likely explanation for the data, given the location of the ligand binding sites [39, 50, 79]; PTH(3-34) binds the N domain and weakly binds the J-domain, and PTH(1-14) analogues bind only to the J-domain.

## IMPLICATIONS OF CLASS B GPCR LIGAND BINDING MECHANISMS IN THE FUTURE DEVELOPMENT OF ALLOSTERIC MODULATORS

Peptide therapeutics have been developed that target Class B GPCR’s (Table **1**), but their use can be complicated by the route of administration, typically injection. For example, Byetta is administered by injection twice daily [19]. In addition, peptides of the size that bind Class B GPCR’s do not readily cross the blood-brain barrier, limiting their use to peripheral disease indications. Allosteric modulation of Class B GPCR’s offers the opportunity of developing low molecular weight, nonpeptide agents that could be administered orally, and that could penetrate the blood-brain barrier to treat CNS disorders. Knowledge of the peptide binding mechanism could prove to be highly valuable in developing such ligands.

Broadly, two types of allosteric modulation could be employed for Class B GPCR’s – allosteric inhibition of peptide binding, and allosteric enhancement. Allosteric inhibitors could be developed as antagonists, targeting the CRF_1_receptor as a potential treatment of stress disorders, the glucacon receptor for managing hyperglycemia, and the CGRP receptor as an alternative mechanism for treating migraine (Table **1**). The two-domain model of peptide binding implies that two different regions of the receptor could be targeted – the J-domain, blocking peptide-stimulated receptor activation (e.g. CRF_1_receptor antagonists), and the N-domain, blocking the principle binding interaction between peptide and receptor. Allosteric enhancers could be developed to potentiate receptor signaling stimulated by the endogenous agonist. Allosteric enhancers of this type have been successfully developed for Class C GPCR’s, e.g. Cinacalcet, an enhancer of the calcium sensing receptor used to treat secondary hyperparathyroidism [82]. The two-domain model could accommodate at least two different enhancer mechanisms – enhancement of peptide affinity for the N-domain, increasing receptor occupancy by the endogenous agonist and so enhancing the signaling output, and enhancement of peptide interaction with the J-domain, directly enhancing the peptide interaction required for receptor activation.

Complexities of ligand binding to Class B GPCR’s could also impact specific stages of drug development. In high-throughput screening using a labeled peptide, the nature of the peptide binding mechanism could affect the outcome. Use of a peptide that binds predominantly to the N-domain could preclude identification of nonpeptide ligands that bind the J-domain. For example, [^125^I] astressin binds to the N-domain of the CRF_1_receptor and is not appreciably displaced by nonpeptide ligands that bind the J-domain [37, 64]. The use of [^125^I]CRF enabled detection of the initial lead compounds because this radioligand binds strongly to the J-domain of the CRF_1_ receptor [38]. In optimizing leads, the nature of the nonpeptide mechanism might need to be considered to define the parameters used to define compound SAR. For example, if a nonpeptide partially blocks peptide binding by a Charniere mechanism, it would be fruitless to attempt to improve maximal inhibition of peptide binding. This parameter is defined by solely by the peptide binding energy for the J-domain, not by an allosteric effect. In selecting compounds to test *in vivo*, the criteria need to be carefully considered. For example, a nonpeptide might only slightly inhibit binding by a Charniere mechanism but could fully antagonize the receptor if the interaction it blocks is required for receptor activation. The use of cellular signaling assays can provide an alternative means to address these issues, from high-throughput screening, through lead optimization and selection for *in vivo* testing.

## CONCLUDING REMARKS

Although the Class B GPCR family is relatively small compared with the Class A family, at least half of the members of the Class B family are attractive therapeutic targets. Allosteric modulation of these receptors represents a potential strategy for the development of low molecular-weight agents as first-generation therapeutics (e.g. for the CRF_1_ receptor) or second-generation alternatives to peptides already in clinical use (e.g. for the GLP-1 receptor). The two-domain interaction of the endogenous peptides with Class B GPCR’s is highly amenable to allosteric modulation by nonpeptide ligands, through modulating the principle binding interaction with the N-domain or the principle activation interaction with the J-domain. Further understanding of these modulatory mechanisms should facilitate the future discovery and optimization of nonpeptide ligands targeting Class B GPCR’s.

## Figures and Tables

**Table 1:** Human Class B GPCR’s and Their Peptide Ligands

Receptor	Peptide Ligand	Principal Biological Action	Major Disease Indication	Peptide Therapeutic	Refs.
CRF_1_	CRF UCN 1	Stress responses Stress responses	Depression (antagonist)		[2,43, 92]
CRF_2_	UCN 1 UCN 2 UCN 3	Stress responses Cardiac contractility Hearing	Heart failure (agonist)		[2]
GHRH	GHRH	Growth hormone release			[52,77]
GIP	GIP	Insulin secretion	Type 2 diabetes (agonist)		[52,77]
Glucagon	Glucagon	Glucose homeostasis	Type 2 diabetes (antagonist)		[52,77]
GLP-1	GLP-1	Insulin secretion	Type 2 diabetes (agonist)	Byetta (Exanatide)	[19,52,77]
GLP-2	GLP-2	Gut mucosal growth			[52,77]
PTH1	PTH PTHrP	Ca^2+^ homeostasis Developmental regulator	Osteoporosis (agonist)	Forteo (PTH(1-34))	[5,58,73, 90]
PTH2	TIP39	Hypothalamic secretion,Nociception			[88]
Secretin	Secretin	Pancreatic secretion			[52,77]
VPAC_1_	VIP PACAP	Neuroendocrine functions Neuroendocrine functions			[30,77]
VPAC_2_	VIP PACAP	Neuroendocrine functions Neuroendocrine functions			[30,77]
PAC_1_	PACAP	Neuroendocrine functions			[30,77]
Calcitonin	Calcitonin	Ca^2+^ homeostasis	Osteoporosis (agonist)	Miacalcin (calcitonin)	[54]
Calcitonin/ RAMP1	CGRP Amylin	Vasodilation Feeding	Migraine (antagonist)		[57,67]
Calcitonin / RAMP3	Amylin	Feeding			[57,67]
CL / RAMP1	CGRP	Vasodilation			[57,67]
CL / RAMP2	Adrenomedullin	Vasodilation			[57,67]
CL / RAMP3	Adrenomedullin CGRP	Vasodilation Vasodilation			[57,67]

Abbreviations:CRF- corticotropin-releasing factor; UCN - urocortin; GHRH - growth hormone-releasing hormone; GIP - glucose-dependent insulinotropic peptide; GLP - glucago nlike peptide;PTH- parathyroidhormone;PTHrP - parathyroid hormone-related protein; TIP39 - tuberoinfundibular peptide of 39 residues; VIP - vasoactive intestinal peptide; PACAP- pituitary adenylate cyclase-activating polypeptide; CGRP - calcitonin gene-related peptide; RAMP - receptor activity modifying protein; CL - calcitonin receptor-like receptor.

## APPENDIX

### Formulation of the Two-Domain Model of Peptide Binding

Peptide binding to Class B GPCR’s can be represented by the two-domain model (Fig. **1A**), in which the carboxyl-terminal portion of the ligand binds the N-domain of the receptor, and the aminoterminal region binds the J-domain [33]. This model assumes peptide ligand (L) binds the receptor N-domain forming RL_N_, defined by the equilibrium association constant *K*_N_. This interaction concentratesL in the vicinity of the J domain, promoting formation of RL_NJ_.This interaction can be described as a reversible isomerization between RL_N_ and RL_NJ_, defined by the isomerization constant *K*_NJ_([RL_NJ_/[RL_N_]).The model assumes interaction between L and the J domain is weak, such that L binding to the J-domain alone (RL_J_) does not contribute significantly to the overall occupancy of receptor by L.

The equilibrium concentration of ligand-bound receptor ([L]_BOUND_)is given by:

(1) $${\left[L\right]}_{\text{BOUND}}=\left[RL_{N}\right]+\left[RL_{\text{NJ}}\right]=\frac{\left[L\right]K_{L}{\left[R\right]}_{\text{TOTAL}}}{1+\left[L\right]K_{L}}$$

where *K*_L_ = *K_N_* (1 + *K_NJ_*) and [R]_TOTAL_ is total receptor concentration ([R]+[RL]_N_+[RL]_NJ_]). The equilibrium concentration of RL_N_, ligand bound only to the N-domain, is given by:

(2) $$\left[RL_{N}\right]=\frac{\left[L\right]K_{N}{\left[R\right]}_{\text{TOTAL}}}{1+\left[L\right]K_{L}}$$

The equilibrium concentration of RL_NJ_, in which L is bound to both the N-domain and J-domain, is given by:

(3) $$\left[RL_{\text{NJ}}\right]=\frac{\left[L\right]K_{N}K_{\text{NJ}}{\left[R\right]}_{\text{TOTAL}}}{1+\left[L\right]K_{L}}$$

Dissociation of L from R is described by eq. 4, derived by assuming L only dissociates fromand isomerization between RL RL_N_ and isomerization between RL_N_ and RL_NJ_ is the dissociation rate constant for L dissociation from is sufficiently rapid to be at steady-state during the dissociation phase. *k*_-N_ is the rate constant for L dissociation from RL_N_ and [L]_BOUND_ = [RL_N_]+[RL_NJ_]

(4) $$\begin{array}{c} \frac{{d\left[L\right]}_{\text{BOUND}}}{\mathrm{dt}}=\left[RL_{N}\right]k_{-N}\\ \left[RL_{N}\right]=\frac{{\left[L\right]}_{\text{BOUND}}}{1+K_{\text{NJ}}}∴\frac{{d\left[L\right]}_{\text{BOUND}}}{\text{dt}}=\frac{{\left[L\right]}_{\text{BOUND}}}{1+K_{\text{NJ}}}k_{-N}\\ \text{By integration}:\\ \begin{array}{c} \frac{{\left[L\right]}_{\text{BOUND},t}}{{\left[L\right]}_{\text{BOUND},t=0}}=e^{-\text{kobs}.t}\\ {\text{where k}}_{\text{obs}}=\frac{1}{1+K_{\text{NJ}}}k_{-N} \end{array} \end{array}$$

Eq. 4 indicates the observed dissociation rate is slowed by isomerization of RL_N_ to RL_NJ_, anticipated since the observed dissociation rate of L from the receptor is first-order with respect to RL_N_.

### Allosteric Modulators that Bind the J-domain of Class B GPCR’s

In this model, formulated in Fig. (**3A**), the modulator M binds a site on the J domain that is spatially distinct from the peptide binding site on the J-domain. Modulator binding does not affect binding of peptide to the N domain but can allosterically modulate peptide binding to the J-domain, defined by the cooperativity constant α.

In Fig. (**3B** and **C**), the effect of M on the binding of L was simulated. Equilibrium binding of a fixed concentration of L was simulated in the presence of a range of concentrations of M (Fig.** 3B**) using eq. 5, derived using the same logic used for eq. 1:

(5) $$\frac{{\left[L\right]}_{\text{BOUND}}}{{\left[L\right]}_{\text{BOUND}}^{\left[M\right]=0}}=\frac{1+[L]K_{L}}{K_{L}}\times \frac{K_{L}+\left[M\right]K_{N}K_{M}\left(1+\alpha K_{\text{NJ}}\right)}{1+\left[L\right]K_{L}+\left[L\right]\left[M\right]K_{N}K_{M}\left(1+\alpha K_{\text{NJ}}\right)+\left[M\right]K_{M}}$$

where [L]_BOUND_ = [RL_N_]+[RL_NJ_]=[MRL_N_]+[MRL_NJ_], ${\left[L\right]}_{\text{BOUND}}^{[M]=0}=\left[{\text{RL}}_{N}\right]+[{\text{RL}}_{\text{NJ}}]$ and *K*_L_ = *K*_N_(1 + *K*_NJ_) (see eq. 1). K_M_ is the equilibrium association constant of M binding to R. In the presence of saturating concentrations of M, binding of L is defined by:

(6) $$\frac{{\left[L\right]}_{\text{BOUND}}^{\left[M\right]\to \infty }}{{\left[L\right]}_{\text{BOUND}}^{\left[M\right]=0}}=\frac{1+[L]K_{N}\;\left(1+K_{\text{NJ}}\right)}{1+[L]K_{N}\;\left(1+\alpha K_{\text{NJ}}\right)}$$

Visual inspection of eq. 6 indicates that a saturating concentration of M does not reduce binding of L to zero when M is an allosteric inhibitor ( α< 1). In addition, modulator can enhance binding of L ( α>1).

In Fig. **(3C**) the effect of M on dissociation of L from the receptor was simulated using eq. 7. This derivation assumes, during the time course of L dissociation, a steady-state of isomerization between RL_N_ and RL_NJ_, and between MRL_N_ and MRL_NJ_, and no disruption of equilibrium between M and the receptor [47]. The derivation also assumes M does not affect the dissociation rate of L from RL_N_.

$\frac{d{\left[L\right]}_{\text{BOUND}}}{\text{dt}}=\left(\left[RL_{N}\right]+\left[\mathrm{MR}L_{N}\right]\right)k_{-N}$

where *k*_-N_ is the dissociation rate constant for L dissociation from RL_N_. The concentration of each L-bound component is given by:

(7) $$\begin{array}{c} \left[{\text{RL}}_{N}\right]=\frac{K_{N}{\left[L\right]}_{\text{BOUND}}}{K_{L}+\left[M\right]K_{N}K_{M}\left(1+{\alpha K}_{\text{NJ}}\right)}\\ \left[{\mathrm{MRL}}_{N}\right]=\frac{\left[M\right]K_{N}K_{M}{\left[L\right]}_{\text{BOUND}}}{K_{L}+\left[M\right]K_{N}K_{M}\left(1+{\alpha K}_{\text{NJ}}\right)}\\ \text{By substitution and re-arrangement}:\\ \frac{d{\left[L\right]}_{\text{BOUND}}}{\text{dt}}=\frac{K_{N}\left(1+\left[M\right]K_{M}\right)k_{-N}}{K_{L}+\left[M\right]K_{N}K_{M}\left(1+{\alpha K}_{\text{NJ}}\right)}{\left[L\right]}_{\text{BOUND}}k_{-N}\\ ∴\frac{{\left[L\right]}_{\text{BOUND},t}}{{\left[L\right]}_{\text{BOUND},t=0}}=e^{-k\text{obs}.t}\\ {\text{where k}}_{\text{obs}}=\frac{K_{N}\left(1+\left[M\right]K_{M}\right)}{K_{L}+\left[M\right]K_{N}K_{M}\left(1+{\alpha K}_{\text{NJ}}\right)}k_{-N} \end{array}$$

In Fig. (**3D** and **E**), the effect of L on the binding of M was simulated. Equilibrium binding of a fixed concentration of M was simulated in the presence of a range of concentrations of L (Fig.3D) using eq. 8:

(8) $$\frac{{\left[M\right]}_{\text{BOUND}}}{{\left[M\right]}_{\text{BOUND}}^{\left[L\right]=0}}=\frac{\left(1+\left[M\right]K_{M}\right)\left(1+\left[L\right]K_{N}\;\left(1+\alpha K_{\text{NJ}}\right)\right)}{1+\left[L\right]K_{L}+\left[M\right]K_{M}\left(1+\left[L\right]K_{N}\left(1+\alpha K_{\text{NJ}}\right)\right)}$$

where [M]_BOUND_ = [MR]+[MRL_N_]+[MRL_NJ_] and ${\left[M\right]}_{\text{BOUND}}^{[L]=0}=[\text{MR}]$ . In the presence of saturating concentrations of L, binding of M is defined by:

(9) $$\frac{{\left[M\right]}_{\text{BOUND}}}{{\left[M\right]}_{\text{BOUND}}^{[L]=0}}=\frac{1+\alpha K_{\text{NJ}}\;+\left[M\right]K_{M}\left(1+\alpha K_{\text{NJ}}\right)}{1+K_{\text{NJ}}\;+\left[M\right]K_{M}\left(1+\alpha K_{\text{NJ}}\right)}$$

Visual inspection of eq. 9 indicates that negative cooperativity (α<1) decreases binding of M, since the numerator is less than the denominator, and reciprocally positive cooperativity (α>1) increases binding of M.

In Fig. (**3E**), the effect of L on dissociation of M was simulated.This derivation makes a number of simplifying assumptions to enable an explicit derivation. As described in ref [47], allosteric modulation of ligand dissociation can result in complex effects on the ligand dissociation curve depending on the relative kinetics of the two ligands. The following derivation assumes that dissociation of L from RL_N_ is much slower (30-fold) than M dissociation from MR, that the kinetics of isomerization between RL_N_ and RL_NJ_ are rapid compared with L dissociation from RL_N_, and that L binding to the J domain accelerates dissociation of M [47]. Under these conditions,MR behaves as a distinct population from the peptide-bound species MRL_N_ and MRL_NJ_. Dissociation of M from MR is described by:

(10) $${\left[\text{MR}\right]}_{t}={\left[\text{MR}\right]}_{t=0}e^{-k_{-M^{.t}}}$$

where *k*_-M_ is the rate of dissociation of M from MR. Dissociation of M from MRL_N_ and MRL_NJ_ is described by:

(11) $$\begin{array}{c} \frac{d\left(\left[{\text{MRL}}_{N}\right]+\left[{\text{MRL}}_{\text{NJ}}\right]\right)}{\text{dt}}=\left[{\text{MRL}}_{N}\right]k_{-M}+{\left[{\text{MRL}}_{\text{NJ}}\right]k}_{-M\left(L\right)}\\ =\frac{\left(\left[{\text{MRL}}_{N}\right]+\left[{\text{MRL}}_{\text{NJ}}\right]\right)\left(k_{-M}+{\alpha K_{\text{NJ}}k}_{-M\left(L\right)}\right)}{1+\alpha K_{\text{NJ}}}\\ ∴{\left(\left[{\text{MRL}}_{N}\right]+\left[{\text{MRL}}_{\text{NJ}}\right]\right)}_{t}={\left(\left[{\text{MRL}}_{N}\right]+\left[{\text{MRL}}_{\text{NJ}}\right]\right)}_{t=0}e^{{-k}_{-M\left(L\right)\text{obs}.t}} \end{array}$$

where *k*_-M(L)_ is the dissociation rate of M from MRL_NJ_ and where:

$k_{-M(L)\text{obs}}=\frac{k_{-M}+\alpha K_{\text{NJ}}k_{-M(L)}}{1+\alpha K_{\text{NJ}}}$

Dissociation of M in the presence of L is the sum of the two exponential equations 10 and 11, as follows:

(12) $$\begin{array}{c} {\left[M\right]}_{\text{BOUND},t}={\left[\text{RM}\right]}_{t=0}e^{-k_{-M^{.t}}}+{\left(\left[{\text{MRL}}_{N}\right]+\left[{\text{MRL}}_{\text{NJ}}\right]\right)}_{t=0}e^{-k_{-M\left(L\right)\text{obs}.t}}\\ ∴\frac{{\left[M\right]}_{\text{BOUND},t}}{{\left[M\right]}_{\text{BOUND},t=0}}=\frac{e^{-k_{-M^{.t}}}+\left[L\right]K_{N}\left(1+{\alpha K}_{\text{NJ}}\right)e^{-k_{-M\left(L\right)\text{obs}.t}}}{1+\left[L\right]K_{N}\left(1+{\alpha K}_{\text{NJ}}\right)} \end{array}$$

### Charniere-Type Model Applied to Class B GPCR’s

In the Charniere model, an antagonist ligand can bind two sites,the first a ‘specific’ site that can be bound by agonist and the second an ‘unspecific’ site that can only be bound by the antagonist [69]. Two separate, hinged regions of the antagonist molecule bind the two distinct sites. This model can be applied to Class B GPCR’s. The formulation (Fig. **3A**) only considers ligand binding,disregarding agonistic or antagonistic properties of the ligands.Full-length peptide ligand (L) binds according to the two-domain model (Fig. **1A**, eqs. 1-4). A second small ligand (A) binds the peptide-binding site on the J domain, defined by the equilibrium association constant *K*_A_. This interaction competitively inhibits peptide binding to the J-domain of the receptor but does not affect peptide binding to the N-domain. Importantly, there is no allosteric interaction between the two ligands in this model.

In Fig. (**4B** and **C**), the effect of A on the binding of L was simulated. Equilibrium binding of a fixed concentration of L was simulated in the presence of a range of concentrations of A (Fig.**4B**) using eq. 13:

(13) $$\frac{{\left[L\right]}_{\text{BOUND}}}{{\left[L\right]}_{\text{BOUND}}^{[A]=0}}=\frac{\left(1+K_{\text{NJ}}\;+\left[A\right]K_{A}\right)\left(1+\left[L\right]K_{L}\right)}{\left[L\right]K_{L}\left(1+K_{\text{NJ}}\;+\left[A\right]K_{A}\right)+\left(1+\left[A\right]K_{A}\right)\left(1+K_{\text{NJ}}\;\right)}$$

where [L]_BOUND_=[RL_N_]+[RL_NJ_]+[RL_N_A_J_], ${\left[L\right]}_{\text{BOUND}}^{[A]=0}=[{\text{RL}}_{N}]+[{\text{RL}}_{\text{NJ}}]$ and *K*_L_ = *K*_N_(1+*K*_NJ_ (see eq. 1). In the presence of saturating concentrations of A, binding of L is defined by:

(14) $$\frac{{\left[L\right]}_{\text{BOUND}}^{[A]\to \infty }}{{\left[L\right]}_{\text{BOUND}}^{[A]=0}}=\frac{1+\left[L\right]K_{L}}{1+\left[L\right]K_{L}+K_{\text{NJ}}}$$

Visual inspection of eq. 14 indicates that saturating concentrations of A do not reduce L binding to zero. Eq. 14 also indicates that A can inhibit binding of L but cannot enhance binding of L, since the denominator is the sum of the numerator and *K*_NJ_.

In Fig. (**4C**) the effect of A on dissociation of L from the receptor was simulated using eq. 15. This derivation assumes, during the time course of L dissociation, a steady-state of isomerization between RL_N_ and RL_NJ_ and no disruption of equilibrium between A and the receptor [47].

$\frac{{d\left[L\right]}_{\text{BOUND}}}{\text{dt}}=\left(\left[{\mathrm{RL}}_{N}\right]+\left[{\mathrm{RL}}_{N}A_{J}\right]\right)k_{-N}$

where *k*_-N_ is the dissociation rate constant for L dissociation from RL_N_. By substitution and re-arrangement:

(15) $$\begin{array}{c} \frac{{d\left[L\right]}_{\text{BOUND}}}{\text{dt}}={\left[L\right]}_{\text{BOUND}}\frac{\left(1+\left[A\right]K_{A}\right)k_{-N}}{1+K_{\text{NJ}}+\left[A\right]K_{A}}\\ ∴\frac{{\left[L\right]}_{{\text{BOUND}}_{t}}}{{\left[L\right]}_{{\text{BOUND}}_{\left(t=0\right)}}}=e^{-k_{{\left(\text{obs}\right)}^{t}}}\\ {\text{where k}}_{\text{obs}}=\frac{1+[A]K_{A}}{1+K_{\text{NJ}}+[A]K_{A}}k_{-N} \end{array}$$

In the presence of saturating concentrations of A, the observed dissociation rate of L is given by:

(16) $$k_{\left(\text{obs}\right)}^{\left[A\right]\to \infty }=k_{-N}$$

Eq. 16 indicates that A accelerates the observed dissociation of L, anticipated since A increases the level of RL_N_ by inhibiting its isomerization to RL_NJ_. Saturating concentrations of ligand A increase the observed rate to that for dissociation from RL_N_ because A competitively inhibits formation of RL_NJ_.

In Fig. (**4D** and **E**), the effect of L on the binding of A was simulated. Equilibrium binding of a fixed concentration of A was simulated in the presence of a range of concentrations of L (Fig.**3D**) using eq. 17:

(17) $$\frac{{\left[A\right]}_{\text{BOUND}}}{{\left[A\right]}_{\text{BOUND}}^{[L]=0}}=\frac{\left(1+\left[A\right]K_{A}\right)\left(1+\left[L\right]K_{N}\right)}{1+\left[A\right]K_{A}\left(1+\left[L\right]K_{N}\right)+\left[L\right]K_{L}}$$

where[A]_BOUND_ =[RA_J_]+[RL_N_A_J_]and ${\left[A\right]}_{\mathrm{BOUND}}^{[L]=0}=[{\mathrm{RL}}_{N}]+[{\mathrm{RL}}_{\mathrm{NJ}}]$ . In the presence of saturating concentrations of L, binding of A is defined by:

(18) $$\frac{{\left[A\right]}_{\text{BOUND}}^{[L]\to \infty }}{{\left[A\right]}_{\text{BOUND}}^{[L]=0}}=\frac{1+\left[A\right]K_{A}}{1+\left[A\right]K_{A}+K_{\text{NJ}}}$$

Visual inspection of eq. 18 indicates that saturating concentrations of L do not reduce A binding to zero. Eq. 18 also indicates that L can inhibit binding of A but cannot enhance binding of A,since the denominator is the sum of the numerator and *K*_NJ_.

In Fig. (**3E**) dissociation of A from the receptor was simulated using Eq. 19:

(19) $$\begin{array}{c} \frac{d{\left[A\right]}_{\text{BOUND}}}{\text{dt}}=\left(\left[{\mathrm{RA}}_{J}\right]+\left[{\mathrm{RL}}_{N}A_{J}\right]\right)k_{-A}\\ ={\left[A\right]}_{\text{BOUND}}k_{-A}\\ ∴\frac{{\left[A\right]}_{\text{BOUND}t}}{{\left[A\right]}_{\text{BOUND}\left(t=0\right)}}=e^{{-k}_{{-A}^{t}}} \end{array}$$

where *k*_-A_ is the dissociation rate constant for A dissociation from RA_J_. From the derivation and Fig. 4A) it is evident that L cannot affect dissociation of A because binding of L to form RL_N_A_J_ does not affect the dissociation rate of A from RA_J_, and RA_J_ cannot isomerize to a different bound state.
